# Comparative gene expression profiling of normal and human colorectal adenomatous tissues

**DOI:** 10.3892/ol.2014.2485

**Published:** 2014-08-27

**Authors:** GONGLIANG DU, XUEHONG FANG, WEI DAI, RUIPENG ZHANG, RUITING LIU, XINGBO DANG

**Affiliations:** 1 Department of Emergency Surgery, Shaanxi Provincial People’s Hospital, Xi’an, Shaanxi 710068, P.R. China; 2 Department of General Surgery, Shaanxi Provincial People’s Hospital, Xi’an, Shaanxi 710068, P.R. China

**Keywords:** colorectal adenoma, gene expression, pathway, gene ontology

## Abstract

Adenomatous colorectal polyps are the precursors of the majority of colorectal cancers. Investigation into the gene expression changes in the progression of colorectal adenoma may offer potential targets for the development of novel diagnostic strategies. Previous gene expression studies have generally been based on a limited number of cases or only focused on a single or a few genes. The present study aimed to identify molecular characteristics of colorectal adenoma through analysis of pathways and gene ontology. The study identified 808 upregulated and 857 downregulated genes. Among the 40 pathways enriched with differentially-expressed genes, the *Staphylococcus aureus* infection pathway and the intestinal immune network for immunoglobulin A production pathway were identified as the most statistically noteworthy pathways at the early stage for colorectal tumorigenesis (P<0.05). These results provide new understanding of colorectal adenoma pathogenesis, with the hope of offering theoretical support for future therapeutic studies.

## Introduction

Colorectal cancer forms by uncontrolled cell proliferation in the colon, rectum or appendix. Genome-scale analysis has indicated that colon and rectal cancers are genetically the same disease since their patterns of genomic alteration are similar (1). Currently, colorectal cancer is the third leading cause of cancer-related mortality (2). The fact that the mortality rate of this disease has decreased in the past few years is attributable to improved treatment and early diagnosis. However, the 5-year survival rate remains at <60% in Europe (3). A high percentage of patients succumb to colorectal cancer every year. Adenomatous colorectal polyps are the precursors of the majority of colorectal cancers (4). Investigation into the gene expression changes in the progression of colorectal adenoma may offer potential targets for the development of novel diagnostic strategies.

Microarray analysis is a powerful approach to investigate transcriptomic changes that may reflect molecular characteristics underlying the pathogenesis of complex diseases. Distinct gene expression patterns in colorectal adenoma have been proposed in previous gene expression studies (5–8). However, these studies were generally based on a limited number of cases or only focused on a single or a few genes. The majority of proteins function through interactions with other proteins in various biological processes. Therefore, pathway- or biological process-based analysis may provide an improved understanding of the mechanism underlying colorectal adenoma progression.

Using a microarray data set from the gene expression omnibus (GEO) database, the present study aimed to identify key deregulated biological processes correlated with colorectal adenoma. Pathways and gene ontology (GO) items with significantly increased dysregulated genes were acquired with the hope of providing novel targets for future molecular diagnostic tests.

## Materials and methods

### Microarray data

Microarray data were collected from the GEO database (http://www.ncbi.nlm.nih.gov/geo/). One dataset (GSE8671) was used for analysis. This dataset contained 32-paired normal mucosa colon and colorectal adenoma biopsy samples. This dataset was based on the GPL570 platform: Affymetrix Human Genome U133 Plus 2.0 Array (Affymetrix, Santa Clara, CA, USA).

### Detection of differentially-expressed genes

Entire data sets, including CEL (the file storing the results of the intensity calculations) and array annotation files for the 64 samples from http://www.ncbi.nlm.nih.gov/geo/query/acc.cgi?acc=GSE8671 were downloaded. The CEL files were generated by Affymetrix DNA microarray image analysis software and contained information regarding all probes. Final quality control of arrays included relative log expression (RLE) and normalized unscaled standard errors from the AffyPLM package (http://www.bioconductor.org/). Arrays showing aberrant RLE plots were excluded from the analysis.

CEL files included raw data generated from satisfactory image files. Raw intensity values from the CEL files were normalized by Robust Multi-array Analysis (RMA) (9) following three steps: First, the effects of background noise and the processing artifacts were neutralized with model-based background correction; second, expression values were aligned to a common scale with quantile normalization; and third, data were summarized and a single expression value for each probe set was generated with an iterative median polishing procedure. The resulting RMA expression value (log_2_-transformed) was derived by probe set level analysis from the raw data of the CEL files.

The log_2_-transformed RMA values for control samples and case samples were stored separately to further identify significantly differentially-expressed genes. Statistical paired t-tests with multiple test correction (Benjamini-Hochberg method) (10) were performed for the case-control to detect differentially-expressed genes. The threshold of significantly expressed genes was set at an FDR of <0.01 and a |fold-change| value of >1 in this study. Differentially-expressed probe sets were identified using fold-change for upregulation or downregulation. Differentially-expressed genes were hierarchically clustered with average linkage and Euclidean distance as a measurement of similarity. All aforementioned procedures were performed using R statistical language (v3.0.1) software (www.r-project.org) with Bioconductor Packages (http://www.bioconductor.org/) (11).

### Pathway enrichment analysis

Selected probes from the Affymetrix Human Genome U133 Plus 2.0 Array were annotated according to the annotation files provided by Affymetrix. All genes were then mapped to Kyoto Encyclopedia of Genes and Genomes pathways (http://www.genome.jp/kegg/) (12). Enrichment analysis was performed by the hyper geometric distribution test to identify pathways significantly enriched with differentially-expressed genes. The observed class was the number of differentially-expressed genes to the total number of genes in each family, while the expected class was the number of all differentially-expressed genes to the total gene number of all families.

### GO analysis

The GO database (http://www.geneontology.org) was generated to address the requirement for consistency when describing gene products. The database was developed to contain three structurally controlled vocabularies, which describe gene products according to their associated biological processes, cellular components and molecular functions. In this study all annotated probes were collected and annotated by gene ontology for three types of functions: Cellular components, molecular functions and biological processes. The enrichment analysis was carried out in a similar way to the pathway enrichment analysis. By using information obtained from the GO database, the present study aimed to identify gene catergories that were overrepresented by differentially expressed genes.

## Results

### Differential expression analyses

Subsequent to quality control, three samples (C13, S11 and S27) were excluded from our analysis due to aberrant RLE plots. Thus, differing from a previous study, which also used the dataset GSE8671, only 29 pairs of arrays were included in the analysis for the detection of differentially-expressed genes and subsequent pathway enrichment analyses (4).

**Figure 1:**
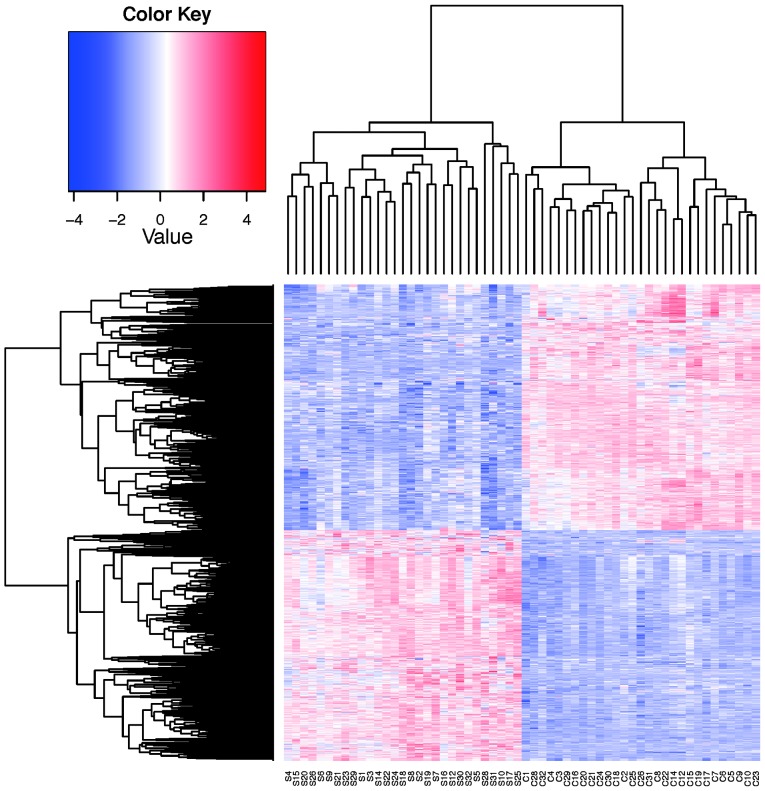
2D hierarchical dendrograms clustered the differentially-expressed genes and studied individuals. The horizontal axis shows the clustering of subjects (S, tumor Samples; C, control; numbers, subject codes), and the vertical axis represents the clustering of the differentially-expressed genes according to their RMA normalized expression intensities.

Compared with normal controls, 1,665 genes were identified as differentially expressed in adenomatous tissues. Among these, 808 exhibited upregulation and 857 exhibited downregulation. Fig. 1 shows the results of the 2D clustering analyses of all the differentially-expressed genes. As shown in Fig. 1, the normal mucosa of the colon samples and the patient colorectal adenoma biopsy can be largely separated into two clusters.

### GO analyses

GO analysis revealed that in the ‘Biological Process’ principle, functions of the differentially-expressed genes are focused on the multicellular organismal process (23.9%) and response to stimulus (23.1%). In the ‘Molecular Function’ principle, the functions are mainly based on protein binding (56.6%). In the ‘Cellular Component’ principle, the products of those genes are primarily located on organelles. As listed in Table I, a total of 18 Gene ontology categories were significantly differentially expressed in adenomas (vs. normal mucosa) with an FDR value of <1.0×10^−6^.

### Pathway analyses

According to the enrichment analysis, 40 pathways were enriched with differentially-expressed genes in adenomatous tissues (P<0.05). As listed in Table II, following correction for multiple comparisons, 19 pathways were identified as being statistically important with regards to colorectal adenomatous carcinogenesis, with an FDR value of <0.01. The top two pathways, the *Staphylococcus aureus* infection pathway and the intestinal immune network for immunoglobulin A (IgA) production pathway were identified as the most statistically noteworthy pathways at the early stage for colorectal tumorigenesis, with an FDR value of <1.0×10^−6^.

## Discussion

The present study reanalyzed 32 pairs of transcriptomic datasets collected from the GEO database in order to characterize the normal mucosa of colon samples and patient colorectal adenoma biopsies (4). By comprehensively examining the differentially-expressed genes and gene sets, and following multiple testing adjustments, the *Staphylococcus aureus* infection pathway and the intestinal immune network for IgA production pathway were highlighted to be in close association with colorectal adenoma.

The intestinal mucosa contains an intact immune system that protects the host from pathogens (13). *Staphylococcus aureus* is a bacterial pathogen that is commonly attached to the human mucosa, and whose secreted proteins and surface components can compromise innate immune responses (14). In the present study, compared with the normal mucosa, the biopsies of colorectal adenoma exhibited downregulated expression of the FCGR2B gene that encodes a type of IgG Fc receptor, FcγRIIB, whose expression has been indicated to be crucial for the regulation of the B cell recall response and the B cell repertoire (15,16). Dysregulated expression of complement cascade-related genes was also found, including genes involved in the classical complement pathways, such as C3 (downregulated), C1S (downregulated), C1QA, C1QB and C1QC (downregulated), and CFI (upregulated), as well as genes involved in alternative complement pathways, such as CFH (downregulated) and CFB (upregulated). The downregulation of C1S, C1Q and C3 (complement components), and the upregulation of CFI (complement component inactivator) indicated the inhibition of the complement system and the susceptibility to bacterial infection (17), while the dysregulation of the CFB and CFH genes indicated the regulation of complement activation (18).

In previous studies, various forms of *Helicobacter pylori* infection were reported to confer an increased risk for colonic neoplasms (19), leading to the understanding that bacteria-induced infection may promote tumorigenesis. To the best of our knowledge, the present study is the first to present the association between *Staphylococcus aureus* infection and colorectal adenoma. However, it cannot be determined whether the correlation indicates that the presence of *Staphylococcus aureus* infection may affect colorectal tumorigenesis or whether colorectal adenoma has an increased susceptibility to *Staphylococcus aureus* colonization, as adenomatous cells may interact with the mucosal immune system (20,21). Further investigation is therefore necessary.

In addition, the present results showed that in the colorectal adenoma biopsy samples, the expression of the majority of genes in the intestinal immune network for IgA production pathway was lower than that in the normal mucosa. IgA is produced in large amounts in the large intestine, and is commonly recognized as the most prevalent antibody in mucosal defense (22,23). It is likely that the impairment of IgA production may drive further inflammatory responses and promote tumor growth. The present study observed that in the IgA production pathway, a total of 22 genes were consistently downregulated. These included a series of human leukocyte antigen (HLA) class II genes (HLA-DOA, DPA1, DPB1, DQA1, DQA2, DQB1, DMB, DRA, DRB1, DRB3, DRB4, and DRB5). These genes encode major histocompatibility complex class II molecules in antigen presenting cells (B lymphocytes, dendritic cells and macrophages), which are important for the proliferation and differentiation of B cells. Additionally, the function of other differentially-expressed genes ranged from T cell activation to B cell development and migration, such as genes CCL28, CXCR4, CXCL12 and ITGA4, which play a significant role in IgA-secreting cell migration. This finding was consistent with a prior study that showed the impaired migration of IgA-secreting cells to colon tumors (20).

In summary, the present results indicated involvement between the *Staphylococcus aureus* infection pathway and the intestinal immune network for IgA production pathway in colorectal adenomatous carcinogenesis. Future validation studies are necessary to clarify the role of mucosal immunity in colorectal cancer.

## Figures and Tables

**Table I:** GO category significantly differentially expressed in adenomas (vs. normal mucosa) with an FDR value of <1.0E-6.

GO category	FDR	Altered genes^a^	Total genes^b^
Cellular component			
Extracellular space	6.45×10^−17^	163	842
Extracellular region	1.51×10^−13^	228	1457
Extracellular matrix	4.48×10^−11^	53	186
MHC class II protein complex	1.44×10^−8^	13	16
Condensed chromosome kinetochore	5.21×10^−8^	26	67
Kinetochore	2.55×10^−7^	25	67
Molecular function			
Chemokine activity	4.87×10^−9^	23	48
MHC class II receptor activity	1.03×10^−7^	12	15
Biological process			
Mitotic cell cycle	1.22×10^−16^	86	321
Immune response	2.54×10^−15^	85	331
M phase of mitotic cell cycle	1.23×10^−13^	39	93
Cell division	6.67×10^−13^	74	293
Mitotic prometaphase	2.64×10^−11^	34	84
DNA replication	4.87×10^−9^	43	148
Mitosis	1.42×10^−8^	49	189
Complement activation, classical pathway	3.23×10^−8^	19	36
Complement activation	1.30×10^−7^	17	31
DNA strand elongation involved in DNA replication	2.39×10^−7^	17	32

a Number of category genes that were significantly upregulated or downregulated in adenomas.

b Number of genes listed in the corresponding GO category.

GO, gene ontology.

**Table II:** KEGG pathway significantly differentially expressed in adenomas (vs. normal mucosa) with an FDR value of <0.01.

Pathway description	Pathway subclass	FDR	Altered genes^a^	Total genes^b^
*Staphylococcus aureus* infection	Infectious diseases	1.17×10^−8^	26	58
Intestinal immune network for IgA production	Immune system	2.92×10^−7^	22	50
Cell cycle	Cell growth and death	4.28×10^−6^	35	123
Asthma	Immune diseases	4.35×10^−6^	16	33
DNA replication	Replication and repair	1.59×10^−5^	16	36
Rheumatoid arthritis	Immune diseases	3.34×10^−5^	27	92
Cell adhesion molecules	Signaling molecules and interaction	3.34×10^−5^	36	143
Graft-versus-host disease	Immune diseases	4.73×10^−5^	17	44
Allograft rejection	Immune diseases	4.98×10^−5^	16	40
Type I diabetes mellitus	Endocrine and metabolic diseases	3.77×10^−4^	16	46
Viral myocarditis	Cardiovascular diseases	4.80×10^−4^	21	73
Complement and coagulation cascades	Immune system	6.13×10^−4^	20	69
Mineral absorption	Digestive system	7.15×10^−4^	16	49
p53 signaling pathway	Cell growth and death	1.45×10^−3^	19	68
One carbon pool by folate	Metabolism of cofactors and vitamins	2.01×10^−3^	9	20
Hematopoietic cell lineage	Immune system	2.20×10^−3^	22	88
Autoimmune thyroid disease	Immune diseases	2.59×10^−3^	16	55
Leishmaniasis	Infectious diseases	4.61×10^−3^	19	75
Cytokine-cytokine receptor interaction	Signaling molecules and interaction	6.79×10^−3^	46	259

a Number of pathway genes that were significantly upregulated or downregulated in adenomas.

b Number of genes listed in the corresponding pathway.

KEGG, Kyoto Encyclopedia of Genes and Genomes; IgA, immunoglobulin A.
